# Comparison of vinorelbine with cisplatin in concomitant chemoradiotherapy in head and neck carcinoma

**DOI:** 10.4103/0971-5851.68845

**Published:** 2010

**Authors:** Devleena, Anup Majumdar, Soumita Poddar, Anindya Chakraborty

**Affiliations:** *Department of Radiotherapy, Chittaranjan National Cancer Institute, Kolkata, India*; 1 *Department of Radiotherapy, Institute of Postgraduate Medical Education and Research, Kolkata, India*

**Keywords:** *Cisplatin*, *concomitant chemoradiotherapy*, *radiosensitizer*, *vinorelbine*

## Abstract

**Aim:**

Head and neck cancer is one of the most commonly occurring malignancies in the world. In India, the most commonly occurring head and neck cancers are those of the oral cavity and the pharynx. The majority of these cancers present with stage III/IV disease. Surgery and radiation therapy are the main treatment modalities. Concomitant chemoradiation is being investigated with the goal of improved local control that translates into improved survival. In this background, we have started this prospective randomized trial to ascertain the dose, schedule and sequence of therapy and to note whether Vinorelbine as radiosensitizer is equally effective as Cisplatin, comparing compliance, local control and toxicity.

**Patients and Methods:**

Forty patients of advanced head and neck cancer were randomized into two arms. Arm A received weekly injection Cisplatin 40mg/m^2^ along with radiation. Arm B received weekly injection of Vinorelbine 6mg/m^2^ along with radiation. Radiotherapy was delivered at a dose of 6,600-7,000 Gy in conventional fractionation in a telecobalt machine.

**Results:**

The complete response (CR) rate was higher in arm B (90%) than in arm A (70%). Major toxicities included neutropenia, anemia, mucositis and nausea.

**Conclusion:**

Concomitant chemoradiation with Vinorelbine produced more CR than chemoradiation with Cisplatin in advanced head and neck cancer. Toxicities were more in the Cisplatin arm, but they were manageable. Although a majority of the study was performed using Cisplatin as the radiosensitizer, Vinorelbine can be recommended as radiosensitizer in advanced head and neck malignancy.

## INTRODUCTION

Head and neck malignancy is the one of the most commonly occurring malignancy in India. The overall male to female ratio is nearly 4:1. It usually occurs in the 5^th^ decade and above. The prognosis of head and neck cancer depends on the primary site, grade and anatomical extent of the disease. Early-stage head and neck cancers can be cured with surgery and/or radiotherapy but, for advanced stages, the local failure rate sometimes approached as high as 50%. To improve the results, combined modality treatment with chemotherapy has been investigated. The three approaches to the use of primary chemotherapy are neoadjuvant chemotherapy,[1–3] adjuvant chemotherapy and concomitant chemoradiotherapy. Concomitant chemoradiation is being investigated with the goals of improved local control translating into improved survival, reduction of distant metastasis and preservation of organ function. The purpose of administering chemotherapy and radiotherapy is to take advantage of the radiosensitizing capability of many of the active drugs for this disease and effect a substantial-enough increase in locoregional control, which would translate to increased survival.[4]

Patients who received concomitant chemoradiotherapy had marginally improved rates of locoregional control and disease-free survival. This was observed primarily in patients with oropharyngeal cancer[5] as compared to other cancers. The drugs most commonly employed as part of a radiation combined approach are Cisplatin, 5FU and hydoxyurea. Cisplatin has widespread use in combined modality treatment in lung cancers[6] and head and neck cancers.[7] Recently, Vinorelbine[8–11] was used as a radiosensitizer. A majority of the studies was performed using Cisplatin[12,13] as a radiosensitizer, although some studies also support use of Vinorelbine as a radiosensitizer.

## PATIENTS AND METHODS

This study was carried out in the radiotherapy department of I.P.G.M.E.R, Kolkata, from September 2004 to July 2005. Forty patients of head and neck cancer were randomized into two arms, with 20 patients in each arm.

Patients of head and neck carcinoma having stage II-IV disease with squamous cell histology were included in this trial. These patients had no prior surgery, chemotherapy or radiotherapy. The performance status was >70% (according to Karnofsky’s scale). Hematological parameters were within the normal range, like hemoglobin >11 mg%, absolute neutrophil count >1,900, platelet count >1 lakh/mm^3^, serum bilirubin <1 mg%, liver enzymes within 1.5-times of the normal limit and serum creatinine <1.5 mg%. Patients were excluded from the study if they had already received some form of anticancer therapy, if there was presence of metastatic disease, if they had participated in a clinical trial in the last 30 days, if there was simultaneous participation in a clinical trial or if they had any uncontrollable systemic illness like diabetes, tuberculosis and hypertension.

### Treatment protocol

Patients who fulfilled the above eligibility criteria were required to sign the informed consent form and were then randomized to assign either of the treatment arms.

Arm A: External beam radiotherapy (EBRT) along with weekly injection Cisplatin 40 mg/m^2^ IV.

Arm B: EBRT along with weekly Vinorelbine 6 mg/m^2^ IV.

The dose of EBRT was 66-70 Gy, with conventional fractionation, using a telecobalt machine with cord sparing after 4,400 cGy.

Response was assessed by local examination and indirect laryngoscopy 1 month after completion of radiotherapy. Regular follow-up was carried out at monthly intervals. Local control was recorded using the terminology complete response (CR), partial response (PR) and progressive disease (PD) (as per WHO definition).

Toxicity assessment was carried out weekly during treatment and thereafter monthly up to 3 months for acute toxicities using Radiation Therapy Oncology Group criteria. Subsequently, patients were being followed-up monthly up to 6 months and then at 3-monthly intervals for any sign of recurrence and treatment-related morbidity.

## RESULTS

### Patient characteristics

From September 2004 to July 2005, 41 patients were enrolled. One patient in arm B dropped out due to mucositis.

Patient characteristics are listed in Table 1. The majority of the patients are in the range of 50–70 years. Patients were predominantly male (95%). They had a good performance status. The larynx and laryngopharynx were the dominant sites (47.5%). Histologically, all were squamous cell carcinoma, the majority of which was well-differentiated (62.5%). Stage III disease was predominant (67.5%). Patients were equally distributed among the two treatment arms.

**Table 1:** Patient characteristics

	Arm A	Arm B
Age		
Median	56.50	62.50
Range	43–70	31–73
Gender		
Male	19	19
Female	01	01
Addiction		
Smoker	15	18
Nonsmoker	05	02
Site		
Laryngopharynx	13	06
Glottis	02	04
Hard palate	00	00
Pyriform fossa	03	03
Tongue	01	04
Tonsil	01	01
Cheek	00	01
Retromol trigone	00	01
Stage			
II	00	02
III	16	11
IV	04	07
Histology		
Well differentiated	05	08
Mod differentiated	14	11
Poor differentiated	01	01

### Response to treatment

All the patients who completed the treatment were assessed in terms of CR, PR, stable disease and PD. Ninety percent of the patients in arm B achieved CR. This result is better than the weekly Cisplatin arm, which has 70% CR (as shown in Table 2).

**Table 2:** Response to treatment

Age	Arm A (RT+Cisp)	Arm B (RT+Vinorelbine)
CR	14	18
PR	04	02
SD	02	00
PD	00	00

When arm B was compared with arm A in terms of CR, it was not statistically significant.

### Acute toxicity

All the toxicities were higher in the Cisplatin-containing arm. All the toxicities were higher in arm A when Cisplatin was used as a radiosensitizer compared with the Vinorelbine arm. Mucositis was almost similar in both arm B and arm A.

When arm B was compared with arm A, myelosuppression was higher in arm A (statistically significant, *P*-value 0.05). Skin reaction was also lower in the Vinorelbine arm when compared with the other arm. Nausea was significantly higher in arm A (RT+cisplatin) when compared with arm B [Table 3.]

**Table 3:** Toxicity

Toxicity	Arm A (RT+Cisplatin)	Arm B (RT+Vinorelbine)
Mucositis	20	19
Skin reaction	14	07
Nausea	16	04
Myelosuppression	13	06

### Late toxicity

As the follow-up is short, no definite comment of late toxicity is possible at this stage. All the patients are alive and no serious complication has occurred till date.

## DISCUSSION

Therapeutic approach in head and neck cancer is widely discussed and is a debatable one also, with the optimum treatment modality, the intention of treatment and managing toxicities occupying the mind of the physician with the survival effect defining the effectivity of treatment modality.

The management of primary cancer is considered separately for each anatomic site. If external beam radiation therapy is selected, it may be given with either conventional once-daily fractionation to 66-70 Gy in 2 Gy/fraction, 5 days a week in a continuous course or with an altered fractionation schedule. EBRT may also be delivered with intensity-modulated radiation therapy (IMRT)[14] to reduce the dose to the normal tissues.[15] The disadvantages of IMRT are that it is much more time consuming to plan and treat the patient, the dose distribution is often less homogeneous so that “hot spots” may increase the risk of late complication and the risk of marginal miss may be increased. Whether an altered fractionation schedule is better than a conventional one depends on the altered fractionation technique that is selected. Altered fractionation schedules shown to result in improved locoregional control rates are the University of Florida hyperfractionation technique and HD Anderson concomitant boost technique. The Randomized Radiation Therapy Oncology Group 90-03 found that acute toxicity is increased with altered fractionation whereas late toxicity is comparable with that of conventional fractionation.

Management of the neck is closely tied to management of the primary site. The rationality of combining chemotherapy with radiation in doses mentioned was:

1. to improve the locoregional control rate and increase the response in this fairly advanced disease
2. assessment of tolerability of patients with a concurrent approach, determining the dose to normal tissues tolerability to avoid toxic effects
3. decrease the distant metastasis rates by acting on systemic micrometastasis present at the diagnosis in more than 50% of the cases.

Calais *et al*,[16] recently reported that disease-free survival and 3-year rate of locoregional control were significantly improved with concomitant chemotherapy, although patients in the combined radiation therapy–chemotherapy arm experienced higher rates of grade 3 or 4 mucositis, feeding tube placement and severe cervical fibrosis.

Although a majority of studies were performed by using Cisplatin as the radiosensitizing drug, some studies also support the use of Vinorelbine as a radiosensitizer.

After 1-year follow-up, CR is higher in the Vinorelbine plus radiation arm followed by the Cisplatin plus radiation arm, which needs further evaluation. Although toxicities like mucosal, hematologic and dermatologic were higher in they concomitant arm, they were manageable. All toxicities were significantly higher when Cisplatin was used as a radiosensitizer. Compliance was also greater with Vinorelbine as toxicities were less when compared with Cisplatin.

Our study had a limited number of patients and the duration of follow-up is also short. Further evaluation of treatment protocol with large number of patients and also with prolonged follow-up may have a positive impact on survival as the response rate is already showing improvement in a concomitant protocol.
